# Safety and efficacy of endovascular treatment for pediatric acute ischemic stroke: a systematic review and Meta-analysis

**DOI:** 10.1007/s11239-025-03227-7

**Published:** 2026-02-09

**Authors:** Hesham Kelani, Mohamed A. Elzayat, Hazem Mohamed Salamah, Ahmed Samir, Munzer Naima, Aesha L. E. Enairat, Ali Dway, Mohammad Hamad, Joshua Singavarapu, Masoom J. Desai, Ahmed Abd Elazim, Volodymyr Vulkanov, Diana Greene-Chandos, David Rosenbaum-Halevi, David P. Lerner, Lisa R. Merlin, Eytan Raz

**Affiliations:** 1 Department of Neurology, SUNY Downstate Health Sciences University at One Brooklyn Health, Brooklyn, NY USA; 2 Faculty of Medicine, Mansoura University, Mansoura, Egypt; 3 Faculty of Medicine, Zagazig University, Zagazig, Egypt; 4 Faculty of Physical Therapy, Cairo University, Giza, Egypt; 5 Faculty of Medicine, University of Aleppo, Aleppo, Syria; 6 Faculty of Graduate Studies, Al-Quds University, Jerusalem, Palestine; 7 Faculty of Medicine, Al-Andalus University for Medical Sciences, Syria, Syria; 8 Faculty of Medicine, University of Jordan, Amman, Jordan; 9 College of Medicine, SUNY Downstate Health Sciences University, Brooklyn, NY USA; 10 Department of Neurology, University of New Mexico, Albuquerque, NM USA; 11 Department of Neurology, University of South Dakota Sanford Medical Center, Sioux Falls, SD USA; 12 Department of Neurology, Rutgers New Jersey School of Medicine, Newark, NJ USA; 13 Department of Neurology School of Medicine, University of Saint Louis, MO, USA; 14 Departments of Neurology, Pharmacology, Physiology, SUNY Downstate Health Sciences University, Brooklyn, NY USA; 15 Department of Neurosurgery, NYU Langone, New York, NY USA

**Keywords:** Acute ischemic stroke, Endovascular, Children, Efficacy, Safety

## Abstract

This meta-analysis aimed at evaluating the safety and efficacy of mechanical thrombectomy (MT) compared to the best medical management (BMM) in children after acute ischemic stroke. We conducted a systematic search of the literature through January 2025. Review Manager Software was used to calculate the odds ratios and their confidence intervals. The primary outcomes of interest were excellent functional recovery (modified Rankin Scale score ≤ 1 (PedmRS 0–1) at 90 days), symptomatic intracranial haemorrhage (sICH), and mortality. A total of eight studies encompassing 8048 patients were included. We found no statistically significant difference between the MT group and the BMM group in terms of mortality (OR 1.17, 95% CI [0.45, 3.05], p-value = 0.75, I^2^ = 0%), sICH (OR 1.32, 95% CI [0.40, 4.39], p-value = 0.6, I^2^ = 0%) and PedmRS 0–1 (OR 1.31, 95% CI [0.68, 2.53], p-value = 0.41, I^2^ = 43%). Furthermore, we performed a subgroup analysis of patients with large vessel occlusion (LVO) stroke and did not find any statistically significant difference between MT and BMM groups in terms of mortality (OR 3.48, 95% CI [0.42, 28.76], p-value = 0.25, I^2^ = 0%), sICH (1.09, 95% CI [0.26, 4.47], p-value = 0.90, I^2^ = 0%) and PedmRS 0–1 (OR 3.62, 95% CI [0.78, 16.76], p-value = 0.10, I^2^ = 59%). However, the BMM group showed increased odds of having a poor functional recovery (PedmRS 3–6) (OR 0.37, 95% CI [0.19, 0.73], p-value = 0.004, I^2^ = 0%). Our findings suggest that both MT and BMM have comparable safety and efficacy outcomes in the pediatric population. Further studies with more strict inclusion criteria are needed to confirm these findings. **PROSPERO Registration: **CRD42024609704.

**Graphical abstract:**

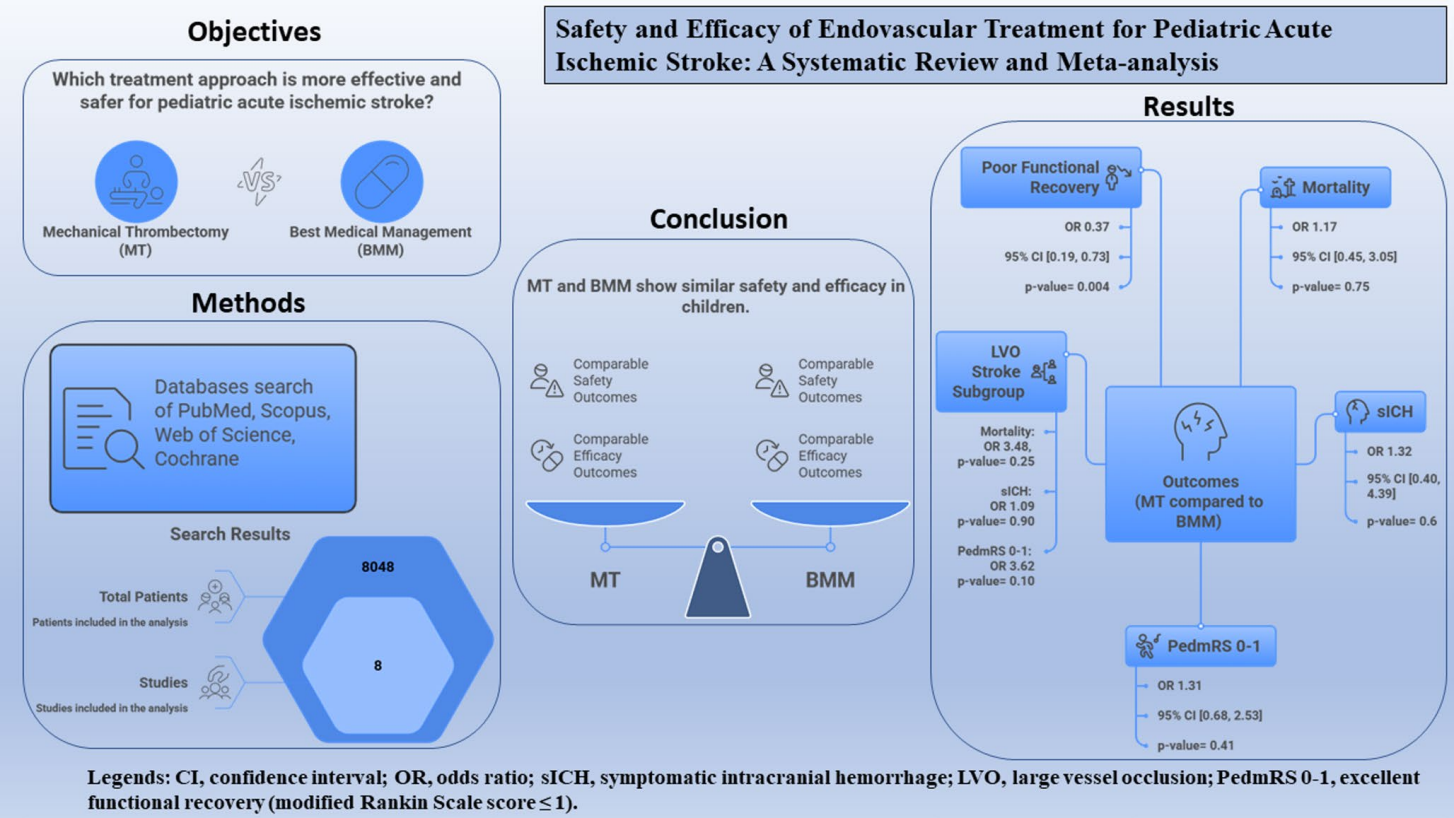

**Supplementary Information:**

The online version contains supplementary material available at 10.1007/s11239-025-03227-7.

## Introduction

Acute ischemic stroke (AIS) represents a significant cause of morbidity and mortality among pediatric populations and contributes to considerable neurological impairments and long-term functional disabilities [1]. AIS affects an estimated 1.3–1.6 per 100,000 children every year in high-income countries [2]. The consequences can be severe, as 70% of pediatric strokes lead to long-term neurological impairments, 20% result in recurrent strokes, and 10% are fatal [3–5].

Pediatric AIS differs substantially from adult stroke in etiology, clinical presentation, and outcomes. In adult stroke patients, one of the most common risk factors is atherosclerosis, which is uncommon in children. Instead, pediatric AIS frequently arises from conditions including cardioembolic causes and arteriopathies [1, 2]. Additionally, clinical presentation in pediatrics can be nonspecific, especially in newborns and younger patients, often presenting with symptoms such as seizures, cardiorespiratory symptoms or headaches, rather than the classic focal neurological deficits found in adult stroke patients [6, 7]. Furthermore, the pediatric brain may also have a greater ability to compensate for ischemic stroke compared to the adult brain, due to its higher neuronal plasticity and potentially more efficient leptomeningeal collaterals, which might affect the outcomes [8, 9].

In adult patients, endovascular therapy has helped to reshape acute ischemic stroke management. In clinical trials, mechanical thrombectomy (MT) significantly improved functional outcomes when performed within a therapeutic up to 24 h [10, 11]. However, the indicated evidence in adult clinical trials cannot be linearly translated into pediatric stroke care due to different etiology, and difficulties in identifying the exact time of ictus in pediatric stroke due to frequent nonspecific presentation. Moreover, the compensation ability of the pediatric brain might reduce the benefit of recanalization therapy [12]. Finally, the small arterial size and the risk of damaging the cerebral vasculature are major concerns when considering MT for pediatric patients [13]. Due to all these reasons, the international guidelines emphasize that MT remains controversial and should be reserved for selected children meeting adult treatment criteria [14, 15].

While there are no prospective randomized clinical trials evaluating the safety and efficacy of recanalization therapy in pediatrics due to small sample size and difficulties in recruitment as observed in the abandoned randomized trial for intravenous thrombolysis [16], several case reports, case series, prospective registry-based and retrospective studies are available in the literature and show positive results [17–19]. However, the sample size of many of these studies is small, and few compared the safety and efficacy of MT vs. best medical management (BMM), and the results are still inconclusive. Therefore, the aim of this systematic review is to assess existing literature on AIS in pediatrics to evaluate and compare the safety and efficacy of endovascular treatment with the best medical management for pediatric stroke.

## Methods

The methodological approach and reporting of the results followed the Preferred Reporting Items for Systematic Reviews and Meta-Analyses (PRISMA) statement guidelines [20, 21]. The review protocol was registered with the International Prospective Register of Systematic Reviews (PROSPERO) database (registration number: CRD42024609704).

### Eligibility criteria

*Participants*: We included children (< 18 years) diagnosed with acute ischemic stroke.

*Intervention*: endovascular thrombectomy (mechanical thrombectomy).

*Comparator*: the best medical management alone (BMM). Intravenous thrombolysis was considered part of the best medical therapy, so it was permitted in both groups.

*Outcomes*: The primary outcomes consisted of excellent functional recovery (modified Rankin Scale score ≤ 1 (PedmRS 0–1) at 90 days), symptomatic intracranial haemorrhage (sICH), and mortality at 90 days. Secondary outcomes included poor functional recovery (modified Rankin Scale score ≥ 3 (PedmRS 3–6) at 90 days), and any intracranial haemorrhage (ICH).

*Study design*: Any cross-sectional, case-control, cohort, clinical trials (RCTs), published in the English language were included. Case reports (< 3 patients) and case series (3–10 patients) were excluded.

### Information sources and search strategy

A comprehensive search was conducted across the following databases: Scopus, Web of Science (WOS), Cochrane Central Register of Clinical Trials (CENTRAL), and MEDLINE via PubMed. The search encompassed articles from inception until September 2024 using the following keywords and their MeSH terms: ischemic stroke, endovascular treatment and children. We further edited our search strategy by adding some more MeSH terms and conducted a second search up to January 2025. Full search strategy can be found in the supplementary file.

### Selection of studies

Before screening the records retrieved from the databases, duplicates were removed using Endnote. Two authors independently evaluated the titles and abstracts using Rayyan software. Relevant studies identified through title and abstract screening were further evaluated using their full-text papers according to the selection criteria. Any disagreements were resolved through consensus or by consulting the first author.

### Data collection

Studies that were included after the full-text screening underwent independent data extraction by two authors. An online Google Sheet was used to collect information, including study ID, population characteristics, intervention details, outcome measures and adverse events. Disagreements were resolved through consensus or by consulting the first authors.

### Risk of bias assessment

Two authors independently assessed the risk of bias using the Risk of Bias in Non-randomized Studies of Interventions tool (ROBINS-I) [22]. The risk of bias was presented using traffic light plots and weighted bar plots generated by the Robvis tool [23].

### Effect estimate

We estimated the odds ratio (OR) and its 95% confidence interval (CI) after endovascular thrombectomy plus the best medical management versus the best medical management alone for the following dichotomous outcomes based on information obtained from the included studies:

- Excellent functional recovery (PedmRS 0–1) at 90 days.
- Symptomatic intracranial haemorrhage (sICH).
- Mortality at 90 days.
- Poor functional recovery (PedmRS 3–6) at 90 days.
- Any intracranial haemorrhage (ICH).

### Synthesis methods

Meta-analysis was conducted using Review Manager Software (RevMan Web) provided by the Cochrane Collaboration. The pooled effect estimate was displayed using a random-effects model and pooled effect estimates are shown within forest plots. Heterogeneity was assessed using the I² statistic, classified as not important (< 40%), moderate (40–60%), substantial (60–75%), and considerable (75–100%) [24]. All results were considered statistically significant at the *p* < 0.05 level.

## Results

### Studies selection

**Fig. 1:**
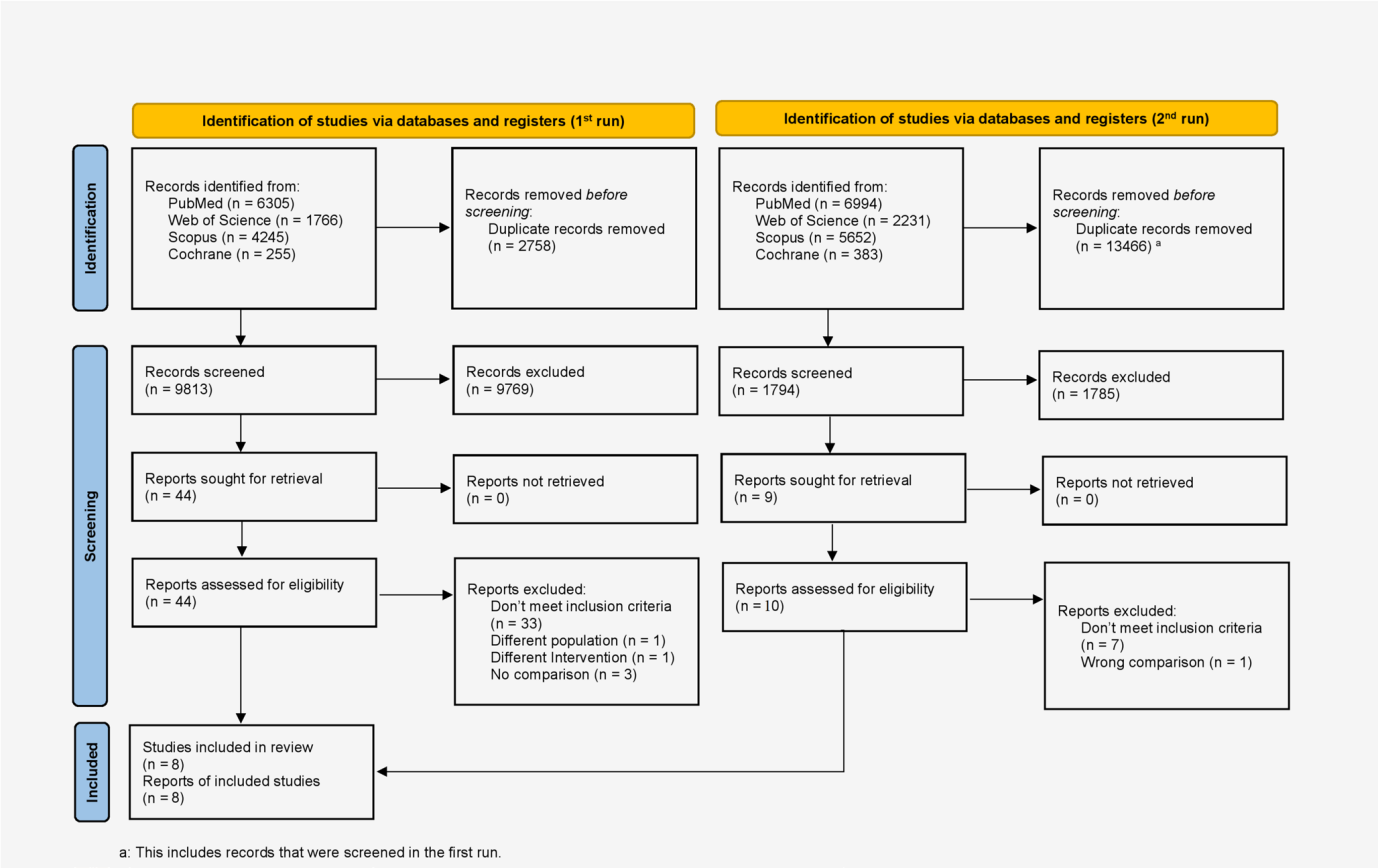
PRISMA flowchart summarizing identification and selection of studies

The initial electronic search across three databases yielded a total of 12,541 studies. After removing duplicates, 9813 studies entered the title and abstract screening phase. Only 44 studies were evaluated for eligibility using their full text. Of those articles, only 6 were found eligible to be included. A second search was conducted and identified 1794 unique publications which were screened first by title and abstract. Only ten were evaluated by full text and only two studies were found eligible, so we included eight studies [25–32] in total as illustrated in Fig. 1.

### Study characteristics

Eight studies, encompassing a total of 8048 participants, with sample sizes ranging from 28 to 7,341 patients, were included in the systematic review. Most studies were retrospective cohort studies, conducted in Europe, with inclusion criteria focusing on patients with ischemic stroke. Bhatia et al., 2023 and Bhatia et al., 2025 was the only study to limit inclusion to patients with large vessel occlusion stroke [25, 32]. A detailed summary of the included studies is presented in Table 1.

**Table 1:** Summary of the included studies

Study ID	Study design	country	Total number of participants	EVT group		Medical management group (*N*)	Primary outcome	sICH definition	Quality assessment
				Number	Techniques				
Kossorotof et al. [28]	Cohort (retrospective)	France	68	40	• Stent Retriever: 19 • Aspiration Thrombectomy: 11 • rescue therapy: 6 • Combined Techniques: 4 • Intravenous tPA only: 4 • Complementary intraarterial thrombolysis: 1	28	Pediatric mRS score 3 months after stroke	According to the ECASS-II definition	Moderate
Bhatia et al. [31]	Cohort (retrospective)	Australia	166	13	• Mechanical thrombectomy: 13	153	Pediatric mRS score 3 months after stroke	Radiologically confirmed acute intracranial hemorrhage associated with acute worsening in the neurological state, specifically an increase in the Pediatric National Institutes of Health Stroke Scale (pedNIHSS) score of 4 or more.	Moderate
Bigi et al. [30]	Cohort (retrospective)	Switzerland	145	6	• Mechanical thrombectomy: 6	139	Complications and PSOM after 6 months	According to the ECASS-III definition	Moderate
Bindslev et al. [26]	Cohort (retrospective)	Denmark	28	14	• Stent Retriever: 7 • Aspiration Thrombectomy: 5 • Combined Techniques: 2	14	mRS	ICH visualized on CT or MRI with progression of neurological symptoms.	Moderate
Dicpinigaitis et al. [29]	Cohort (retrospective)	USA	7341	190	• Mechanical thrombectomy: 190	7151	functional outcome evaluated using patient discharge disposition	NA	Moderate
Bhatia et al. [25]	Case-control	Australia and Canada	52	26	• Aspiration Thrombectomy: 3 • Stent Retriever: 6 • Combined Techniques:17 • Balloon angioplasty: 1 • Intravenous tPA only: 1^**a**^	26	Pediatric mRS score 3 months after stroke	According to the ECASS-III definition	Moderate
Sporns et al. [27]	Cohort (prospective)	Europe, North America, South America, Asia, and Australia	208	117	• Aspiration Thrombectomy: 31 • Stent Retriever: 72 • Combined Techniques:14	91	difference in modified Rankin Scale (mRS) score between baseline and 90 days	An increase in the PedNIHSS score of at least 4 points with presence of parenchymal haemorrhage.	Moderate
Bhatia et al. [32]	Case-control	Multicenter	40	24	• Stent-retriever: 17 • Aspiration: 7	16	Pediatric mRS	Intracranial extravascular blood associated with a ≥ 4-point increase in the pediatric National Institutes of Health Stroke Scale (Ped-NIHSS) score.	Moderate

a: two patients had recurrent occlusion in the same angiographic location within 96 h and underwent a second thrombectomy procedure. One patient who received bridging intravenous tPA en route to the angiography suite after consenting to mechanical thrombectomy had successful recanalization on the first angiographic run and received zero passes

Of the 8048 participants, 430 were treated with mechanical thrombectomy (MT) while the remaining 7618 were treated with the best medical management. Regarding the MT group, 177 (42%) patients were females. The mean age ranged from 10.3 years to 13.7 years and the mean NIHSS ranged from 11.4 to 15.3. Furthermore, 132 patients (31%) received IVT together with MT. On the other hand, 3334 (44.6%) patients were females in the best medical management group. The mean age ranged from 5.6 years to 11 years while the mean NIHSS ranged from 4.8 to 14.2. Furthermore, 215 patients (2.8%) received IVT as a part of their care (Table 2).

**Table 2:** Baseline characteristics of the included studies

Study ID		Number of participants	Age, years, (mean ± SD)	Sex (Female) N (%)	Baseline NIHSS, (mean ± SD)	Stroke location						Stroke etiology					IV Thrombolysis N (%)
						ICA	ACA	MCA	PCA	Vertebra−basilar	Tandem occlusion	Cardio−embolic	Arteriopathy	coagulopathy	Idiopathic	other	
Kossorotof et al. [28]	EVT group	40	10.3 ± 9.2	15 (37.5)	15.3±7.7	Anterior circulation: 33 (82.5)			Posterior circulation: 7 (17.5)		5/34 (14.7)	14 (35)	18 (45)	4 (10)	3 (7.5)	1 (2.5)	16 (40)
	Medical management	28	11 ± 8.6	9 (32.1)	10.7 ± 8.6	Anterior circulation: 24 (85.7)			Posterior circulation: 4 (14.2)		1/19 (5.3)	7 (25)	11 (39.3)	4 (14.3)	5 (17.9)	1 (3.6)	28 (100)
Bhatia et al. [31]	EVT group	13	12.4 ± 4.4	3 (23.1)	NA	NA	NA	NA	NA	NA	NA	6 (46.2)	3 (23.1)	1 (7.7)	2 (15.4)	1 (7.7)	3 (23.1)
	Medical management	153	5.6 ± 5.1	53 (41.7)	NA	NA	NA	NA	NA	NA	NA	45 (29.4)	42 (27.5)	2 (1.3)	30 (19.6)	34 (22.2)	1 (0.7)
Bigi et al. [30]	EVT group	6	12.5 ± 5.1	NA	14.7 ± 6.9	3 (50)	0	2 (33.3)	0	1 (16.7)	0	NA	NA	NA	NA	NA	1 (16.7)
	Medical management	139	5.8 ± 4.8	NA	8.3 ± 4.9	17 (12.2)	0	94 (67.6)	10 (7.2)	13 (9.4) a	0	NA	NA	NA	NA	NA	5 (3.6)
Bindslev et al. [26]	EVT group	14	11.7 ± 5.8	4 (28.6)	12 ± 6.2	2 (14.3)	0	3 (21.4)	1 (7.1)	7 (50)	1 (7.1)	4 (28.6)	8 (57.1)	0	1 (7.1)	2 (14.3)	3 (21.4)
	Medical management	14	5.7 ± 4.9	6 (42.9)	NA	2 (14.3)	0	8 (57.1)	2 (14.3)	0	2 (14.3)	3 (21.4)	6 (42.9)	0	2 (14.3)	3 (21.4)	0
Dicpinigaitis et al. [29]	EVT group	190	13.7 ± 4.1	79 (41.8)	11.4 ± 5.9	15 (7.9)	NA	NA	NA	25 (13.2)	NA	30 (15.8)	51 (26.8)	20 (10.5)	NA	NA	79 (41.6)
	Medical management	7151	8.4 ± 8.5	3214 (44.9)	4.8 ± 5.8 **b**	290 (4.1)	NA	NA	NA	158 (2.2)	NA	1251 (17.5)	702 (9.8)	466 (6.5)	NA	NA	154 (2.2)
Bhatia et al. [25]	EVT group	26	10.9 ± 4.4	10 (38.5)	11.8 (range: 0−22)	10 (38.4)	0	14 (53.8)	0	2 (7.7)	0	14 (53.9)	4 (15.4)	1 (3.9)	5 (19.2)	2 (7.7)	4 (15.4)
	Medical management	26	9.8 ± 4.4	11 (42.3)	NA	10 (38.4)	0	14 (53.8)	0	2 (7.7)	0	8 (30.8)	14 (53.9)	1 (3.9)	3 (11.5)	0	1 (3.8)
Sporns et al. [27]	EVT group	117	10.3 ± 6	55 (47)	14.3 ± 6.8	37/127 (29.1)	5/127 (3.9)	65/127 (51.1)	3/127 (2.4)	17/127 (13.4)	NA	51 (43.6)	27 (23.1)	NA	NA	NA	20 (17.1)
	Medical management	91	7 ± 6.8	38 (42)	9 ± 6.03	16/97 (16.5)	3/97 (3.1)	64/97 (66)	8/97 (8.3)	5/97 (5.2) **c**	NA	15 (16.5)	47 (51.6)	NA	NA	NA	25 (27.5)
Bhatia et al. [32]	EVT group	24	9.4 ± 5.4	11 (45.8)	14.4 ± 5.5	8/24 (33.3)	0	16/24 (66.7)	0	0	NA	12 (50)	NA	NA	NA	NA	6 (25)
	Medical management	16	8.5 ± 5.2	3 (18.8)	14.2 ± 8.9	8/16 (50)	0	8/16 (50)	0	0	NA	5 (31)	NA	NA	NA	NA	1 (6)

a: five (3.6%) patients had occlusion in SCA/PICA

b: NIHSS was recorded for 70 patients treated with EVT and 380 patients treated with medical management

c: one patient had an occlusion in the Superior proximal superior cerebellar artery

### Risk of bias assessment

The eight studies had an overall moderate risk of bias [25–32]. Notably, all studies had a moderate risk of bias in the 1 st two domains of the ROBINS-I tool: bias due to confounding and bias due to selection of participants (Figs. S1and S2) [1]. 

### Outcomes in pediatrics with acute ischemic stroke

#### Mortality

**Fig. 2:**
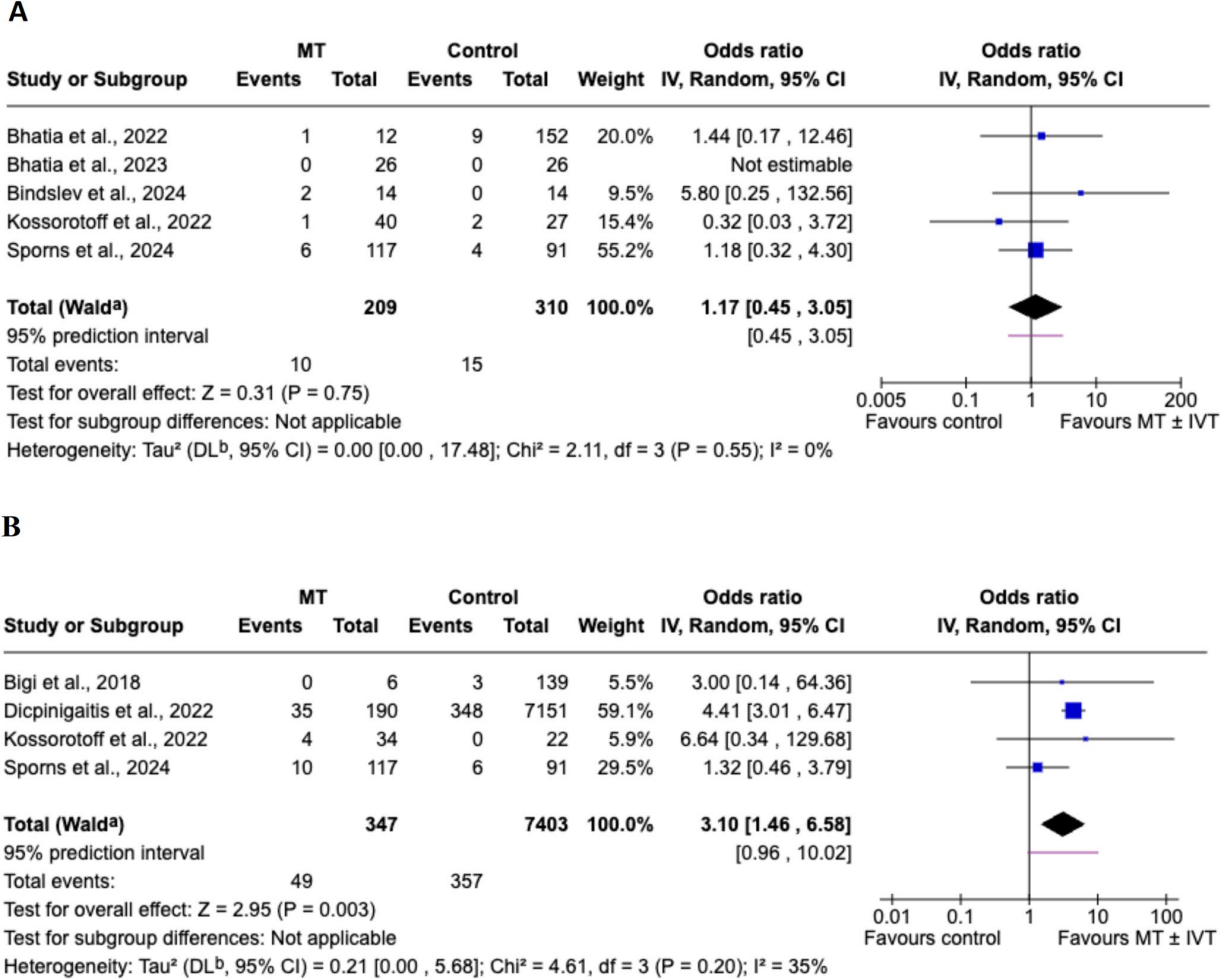
Forest plots for (**a**) mortality in patients with acute ischemic stroke and (**b**) any ICH in patients with acute ischemic stroke

Four studies reported mortality in 90 days with 209 patients in the MT group and 310 in the best medical management. No statistically significant difference was observed between the two groups (OR 1.17, 95% CI [0.45, 3.05], p-value = 0.75). The pooled studies were found homogenous (I^2^ = 0%, p-value = 0.55) (Fig. 2a).

#### Any ICH

Four studies reported ICH after treatment, including 347 patients in the MT group and 7403 in the best medical management group. The mechanical thrombectomy group showed statistically significant increased odds of intracranial hemorrhage compared to the best medical management group (OR 3.10, 95% CI [1.46, 6.58], p-value = 0.003). Low heterogeneity was observed across the pooled studies (I^2^ = 35%, p-value = 0.2) (Fig. 2b).

#### Symptomatic ICH

**Fig. 3:**
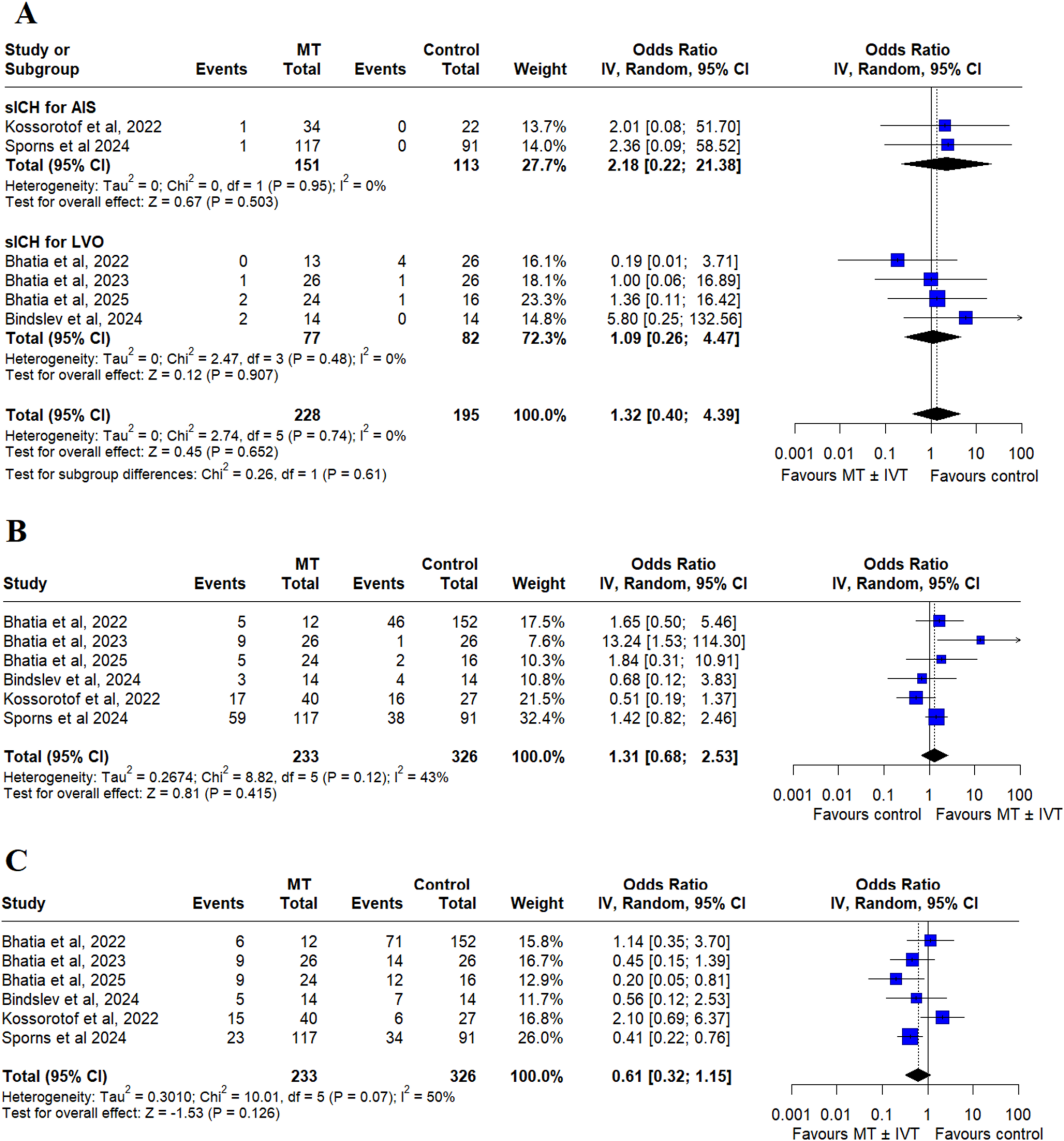
Forest plots for (**a**) symptomatic ICH in patients with acute ischemic stroke and large vessel occlusion stroke, (**b**) excellent functional recovery in patients with acute ischemic stroke, and (**c**) poor functional recovery in patients with acute ischemic stroke

Seven studies, including 228 patients in the MT group and 195 in the best medical management group, reported symptomatic ICH after treatment. However, the Bigi et al. study reported no sICH in both groups and therefore did not contribute to the meta-analysis [30]. There was no statistically significant difference between the two groups (OR 1.32, 95% CI [0.40, 4.39], p-value = 0.65). No heterogeneity was found across the pooled studies (I^2^ = 0%, p-value = 0.7) (Fig. 3a).

#### Excellent functional recovery

Six studies reported excellent recovery at 90 days after stroke (PedmRS 0–1), including 233 in the MT group and 326 in the best medical management group. No statistically significant difference was observed between the two groups (OR 1.31, 95% CI [0.68, 2.53], p-value = 0.41). Moderate heterogeneity was observed across the pooled studies (I2 = 43%, p-value = 0.12) (Fig. 3b).

#### Poor functional recovery

Six studies reported poor functional recovery 90 days after stroke (PedmRS 3–6), including 233 in the MT group and 326 in the best medical management group. There was no statistically significant difference between the two groups (OR 0.61, 95% CI [0.32, 1.15], p-value = 0.13). Moderate heterogeneity was observed across the pooled studies (I^2^ = 50%, p-value = 0.07) (Fig. 3c).

### Outcomes in pediatrics with large vessel occlusion stroke

#### Mortality

Three studies reported mortality in 90 days among patients with LVO stroke, but Bhatia et al. (2023) study reported no mortality in 90 days and therefore only two studies were included in the meta-analysis [25]. There was no statistically significant difference between the two groups (MT and the best medical management) (OR 3.48, 95% CI [0.42, 28.76], p-value = 0.25). In addition, no heterogeneity was found across the pooled studies (I^2^ = 0%, p-value = 0.66) (Fig. S3).

#### Symptomatic ICH

Three studies out of the six that reported sICH were reporting it among patients with LVO, so we performed a subgroup analysis based on the type of stroke (Fig. 3a). No statistically significant difference was observed between the two groups (OR 1.09, 95% CI [0.26, 4.47], p-value = 0.90). Low heterogeneity was observed across the three studies (I^2^ = 0%, p-value = 0.48).

#### Excellent functional recovery

Three studies reported excellent recovery at 90 days after stroke (PedmRS 0–1), including 76 in the MT group and 82 in the best medical management group. No statistically significant difference was found between the two groups (OR 3.62, 95% CI [0.78, 16.76], p-value = 0.10). Moderate heterogeneity was observed across the pooled studies (I^2^ = 59%, p-value = 0.06) (Fig. S4a).

#### Poor functional recovery

Three studies reported poor functional recovery at 90 days after stroke (PedmRS 3–6), including 76 in the MT group and 82 in the best medical management group. The best medical management group showed statistically significant increased odds of having a poor functional recovery (OR 0.37, 95% CI [0.19, 0.73], p-value = 0.004). The pooled studies were found homogenous (I^2^ = 0%, p-value = 0.77) (Fig. S4b).

Heterogeneity was investigated for possible causes through subgroup analysis based on large vessel occlusion and was resolved only in the poor functional recovery group. Other possible causes of heterogeneity were discussed in the discussion section. Sensitivity analysis was conducted to show the effect of the fixed-effect model on the robustness of the results, and no significant effects were found for any outcomes.

## Discussion

Our meta-analysis results showed an insignificant difference between MT and BMM in pediatric AIS regarding safety and efficacy outcomes, except for hemorrhagic outcomes, where MT demonstrated increased odds of intracranial haemorrhage compared to BMM. Furthermore, in cases of large vessel occlusion, the best medical management showed poorer functional recovery than MT, as indicated by the PedmRS scores of 3–6. However, the current evidence should be interpreted cautiously because there were no randomized controlled trials (RCTs). However, it’s noteworthy that conducting an RCT on this topic is difficult, as discussed in the introduction.

While MT is used for large vessel occlusion stroke, most of the included studies focused on acute ischemic stroke in children, irrespective of stroke location. This might be due to the low incidence of ischemic stroke in children, which makes it difficult to find enough cases with LVO stroke. Notably, this comparison may disproportionately favor BMM, as small vessel occlusion (SVO) strokes are generally associated with better prognosis due to small infarct size [33]. This can explain why there was no difference between MT and BMM regarding functional outcomes when the comparison included all patients with AIS. Only when comparing outcomes of LVO stroke, the BMM showed poorer functional recovery. This finding is in line with a meta-analysis of RCTs in adults with AIS, predominantly involving LVO cases, where BMM significantly elevated the risk of unfavorable functional outcomes [34].

Regarding 90-day mortality, we found no difference between the two groups when including all AIS patients or LVO stroke patients. This is different from adult data, which shows that MT significantly decreased the risk of mortality compared with BMM [34]. This difference may be due to differences in etiology between adults and the pediatric population [1, 2]. In addition, as the MT devices are not designed for the small pediatric vasculature, this carries the risk of damaging the vasculature, which may be fatal [13].

Regarding hemorrhagic outcomes, we found that MT is associated with increased odds of any ICH, which is similar to a recently published systematic review of RCTs about acute ischemic stroke with a large infarct area [35]. However, this observation may be prognostically inconsequential as we didn’t find statistically significant differences between the two groups in terms of mortality and functional outcomes when including all patients with AIS. Furthermore, MT showed better functional recovery than the BMM in patients with LVO stroke. In addition, we found no difference in symptomatic ICH when including all AIS patients or LVO patients, which further indicates that this hemorrhage is not severe enough to be symptomatic. Similar findings were observed in the meta-analysis of RCTs in adults with AIS, mainly LVO, which observed no significant difference regarding symptomatic ICH between MT and BMM groups [34].

A similar meta-analysis was recently published, examining the efficacy of MT versus medical treatment [36]. They found that MT was significantly associated with better function outcome (mRS 0–1 and mRS 0–2). However, they missed four studies that are included in our study. Furthermore, they included a case series study which may produce a significant bias.

Some heterogeneities were found in the analyses. First, in any ICH variable, we found low heterogeneity which can be a result of the very large sample size of the Dicpinigaitis et al. (2022) study compared to other studies [29]. In addition, in the subgroup analysis of symptomatic ICH for LVO stroke, low heterogeneity was also found which can be attributed to differences in symptomatic ICH definition among the included studies. Notably, Bindslev et al. (2024) study had the vaguest definition as they didn’t count on the NIHSS score in their definition [26]. Functional outcomes also showed heterogeneity either in AIS in general or the LVO group. This can be attributed to inconsistency in the time of assessment. While all studies reported PedmRS at 90 days, Sporns et al. (2024) study reported it at 90 days (± 10 days) [27] and Bindslev et al. (2024) study reported it at 3 months (IQR 2.5–3.9 months) [26].

### Limitations

Our systematic review has some limitations; first, the included studies are observational as no RCTs were found, which introduces bias related to confounding variables, participant selection, and selective reporting of results, as indicated by the risk of bias assessment results. Second, many studies compared the outcomes in patients with acute ischemic stroke, not LVO stroke which might make the results biased towards the medical treatment. However, we performed a subgroup analysis for patients with LVO stroke. Third, due to the non-availability of sufficient data, we couldn’t perform a subgroup analysis based on stroke severity, occlusion site and age. Fourth, the mean age of the children in the studies ranged from 5.6 to 13.7 years, limiting the results’ applicability to infants and toddlers. Similarly, all the included studies were conducted in high-income countries, so the outcomes and applicability to middle- and low-income countries may be limited. Finally, we searched for English-language studies only which might introduce a language bias. However, Morrison et al. (2012) found no difference in treatment effect between systematic reviews that used English and those that included studies in languages other than English [37].

### Implications for future research

Future research should focus on comparing the safety and efficacy of MT vs. medical treatment in children with LVO strokes in larger samples to help in controlling different confounders such as stroke severity, age and etiology. Furthermore, investigating the treatment effect in different etiologies is still needed especially in focal cerebral arteriopathy (FCA) as previous studies found a potential selection bias based on etiology. It was noted that patients with cardioembolic causes were more likely to be treated with MT compared to FCA indicating a bias by perceived feasibility [27, 38]. In addition, our analysis shows increased odds of ICH in the MT group without differences between the two groups in mortality, symptomatic ICH and functional outcomes. This suggests that the haemorrhage may not be severe enough to impact the outcomes but the reasons for this observation need further investigation. Finally, Long-term outcomes, such as the one-year Ped mRS, need to be addressed in future research.

## Conclusion

MT shows safety and efficacy outcomes comparable with the best medical management (BMM) in pediatric patients with AIS. Only ICH was found to be higher in the MT group with no effect on mortality or functional outcomes. In patients with LVO stroke, patients treated with BMM showed poorer functional outcomes (PedmRS 3–6) at 90 days compared to the MT group highlighting the superiority of MT in these patients. Future research should expand studies to diverse populations and focus on long-term outcomes. Improved endovascular techniques tailored to pediatric vasculature are also needed to optimize safety and efficacy.

## Supplementary Information

Below is the link to the electronic supplementary material.

Supplementary Material 1

## Data Availability

All data generated or analyzed during this work are included in this article and its supplementary materials.
