# Supplementary material for: Safety and efficacy of endovascular treatment for pediatric acute ischemic stroke: a systematic review and Meta-analysis
Source: J Thromb Thrombolysis. 2026 Feb 9;59(5):1241–54. doi: 10.1007/s11239-025-03227-7 (PMC13331862; doi:10.1007/s11239-025-03227-7)
Supplement: Supplementary file 1 — Supplementary Material 1 [file 11239_2025_3227_MOESM1_ESM.docx]

**Safety and Efficacy of Endovascular Treatment for Pediatric Acute Ischemic Stroke: A Systematic Review and Meta-analysis**

Hesham Kelani^1⁋^, Mohamed A. Elzayat^2 ⁋^, Hazem Mohamed Salamah^3^. Ahmed Samir^4^, Munzer Naima^5^, Aesha L.E Enairat^6^, Ali Dway^7^, Mohammad Hamad^8^, Joshua Sinavarapu^9^, Masoom J. Desai^10^, Ahmed Abd Elazim^11^, Volodymyr Vulkanov^12*^, Diana Greene-Chandos^13^, David Rosenbaum^1^, David P. Lerner^1^, Lisa R. Merlin^1,14^, Eytan Raz^15^

1. Department of Neurology, SUNY Downstate Health Sciences University at One Brooklyn Health, Brooklyn, NY.
2. Faculty of Medicine, Mansoura University, Mansoura, Egypt. ORCID ID: (0000-0003-1141-2484)
3. Faculty of medicine, Zagazig University, Zagazig, Egypt.
4. Faculty of Physical Therapy, Cairo University, Giza, Egypt.
5. Faculty of Medicine, University of Aleppo, Aleppo, Syria.
6. Faculty of Graduate Studies, Al-Quds University, Jenin, Palestine.
7. Faculty of Medicine, Al-Andalus University for Medical Sciences, Syria.
8. Faculty of Medicine, University of Jordan, Amman, Jordan.
9. School of Medicine, SUNY Downstate Health Sciences University, Brooklyn, NY.
10. Department of Neurology, University of New Mexico, Albuquerque, NM.
11. Department of Neurology, University of South Dakota Sanford Medical Center, Sioux Falls, SD.
12. Department of Neurology, Rutgers New Jersey School of Medicine, Newark, NJ, USA.
13. Department of Neurology, School of Medicine, University of Saint Louis, MO.
14. Departments of Neurology, Pharmacology, Physiology, SUNY Downstate Health Sciences University, Brooklyn, NY.
15. Department of Neurosurgery, NYU Langone, New York, NY.

⁋ Hesham Kelani and Mohamed A. Elzayat contributed equally to this work.

*Correspondence

Volodymyr Vulkanov

Affiliation: Department of Neurology, Rutgers New Jersey School of Medicine, Newark, NJ, USA.

Emsil: [vv263@njms.rutgers.edu](mailto:vv263@njms.rutgers.edu)

**Search strategy**

***Cochrane:***

#1 "cerebrovascular disorders" OR "basal ganglia cerebrovascular disease" OR "brain ischemia" OR "carotid artery diseases" OR "carotid artery thrombosis" OR "carotid artery, internal, dissection" OR "intracranial arterial diseases" OR "cerebral arterial diseases"

#2 "anterior cerebral artery infarction" OR "middle cerebral artery infarction" OR "posterior cerebral artery infarction" OR "infarction, anterior cerebral artery" OR "infarction, middle cerebral artery" OR "infarction, posterior cerebral artery"

#3 "Lacunar Infarct" OR "Lacunar Infarction" OR "intracranial embolism" OR "intracranial thrombosis" OR "brain infarction" OR "vertebral artery dissection"

#4 ((brain OR cerebr* OR cerebell* OR vertebrobasil* OR hemispher* OR intracran* OR intracerebral OR infratentorial OR supratentorial OR middle cerebr* OR anterior circulation) near/5 (Ischemi* OR Ischaemi* OR infarct* OR thrombo* OR emboli* OR occlus* OR hypoxi*))

#5 ((Ischemi* OR Ischaemi*) near/6 (stroke* OR apoplex* OR cerebral vasc* OR cerebrovasc* OR cva OR attack*))

#6 "cerebral sinus thrombosis" OR "cerebral venous sinus thrombosis" OR "CVST" OR "CVT"

#7 #1 OR #2 OR #3 OR #4 OR #5 OR #6

#8 "interventional radiology" OR catheterization OR "balloon catheterization" OR angioplasty OR "laser‐assisted balloon angioplasty" OR "laser angioplasty" OR atherectomy OR "catheter ablation" OR embolectomy OR "aspiration embolectomy" OR "balloon embolectomy"

#9 thrombectomy OR "mechanical thrombectomy" OR neurothrombectom* OR "thrombus aspiration" OR "blood vessel prosthesis" OR "blood vessel transplantation" OR "cerebral revascularization" OR reperfusion OR dilatation

#10 angioplast* OR stent* OR atherect* OR thromboaspiration OR endoluminal repair* OR endovascular snare* OR neuronet OR microsnare OR angiojet OR "penumbra system" OR "solitaire" OR "trevo"

#11 (interventional NEAR/3 (radiolog* OR radiograph* OR neuroradiolog*))

#12 ((mechanical OR radiolog* OR pharmacomechanical OR laser OR endovascular OR neurovascular) NEAR/5 (thrombolys* OR reperfusion OR fragmentation OR aspiration OR recanalisation OR recanalization OR clot lysis OR clot lyses))

#13 ((clot OR thrombus OR thrombi OR embol*) NEAR/5 (aspirat* OR remov* OR retriev* OR fragmentation OR retract* OR extract* OR obliterat* OR dispers*))

#14 ((retrieval OR extraction) NEAR/5 device*)

#15 (("blood vessel" OR artery) NEAR/5 (prosthesis OR implantat*))

#16 ((merci OR concentric) NEAR/5 retriever)

#17 ((Endovascular OR intravascular) NEXT (Procedure* OR Technique* OR treatment*))

#18 #8 OR #9 OR #10 OR #11 OR #12 OR #13 OR #14 OR #15 OR #16 OR #17

#19 child OR children OR pediatric* OR paediatric* OR teen* OR Adolescen* OR Youth* OR newborn* OR neonate* OR toddler* OR infant*

#20 "below 18 years" OR "under 18 years" OR "younger than 18"

#21 #19 OR #20

#22 #7 AND #18 AND #21

***PubMed:***

#1 "Ischemic Stroke"[Mesh] OR stroke OR "acute stroke*" OR "ischemic stroke" OR "acute ischemic stroke" OR " Embolic Stroke" OR " Thrombotic Stroke" OR "cerebrovascular Accident*" OR "cerebral vascular accident*" OR "Brain Vascular Accident*" OR "cerebrovascular disorders" OR "basal ganglia cerebrovascular disease" OR "thrombotic events" OR "thromboses"

#2 "acute cerebral ischemia" OR "cerebral ischemia" OR "brain ischemia" OR "cerebral infarction" OR "brain infarction" OR "Lacunar Infarct" OR "Lacunar Infarction" OR "intracranial embolism" OR "intracranial thrombosis" OR "vertebral artery dissection"

#3 "carotid artery diseases" OR "carotid artery thrombosis" OR "carotid artery, internal, dissection" OR "intracranial arterial diseases" OR "cerebral arterial diseases" OR "cerebrovascular apoplex*"

#4 "cerebral arterial occlusion" OR "cerebral artery occlusion" OR "anterior cerebral artery infarction" OR "middle cerebral artery infarction" OR "posterior cerebral artery infarction" OR "infarction, anterior cerebral artery" OR "infarction, middle cerebral artery" OR "infarction, posterior cerebral artery" OR "cerebral sinus thrombosis" OR "cerebral venous sinus thrombosis" OR "CVST" OR "CVT"

#5 #1 OR #2 OR #3 OR #4

#6 "Endovascular Procedures"[Mesh] OR "endovascular Procedure*" OR "endovascular Technique*" OR "endovascular treatment*" OR "endovascular therap*"

#7 angioplasty OR "laser‐assisted balloon angioplasty" OR "laser angioplasty" OR atherectomy OR "catheter ablation" OR embolectomy OR "aspiration embolectomy" OR "balloon embolectomy" OR "interventional radiology" OR catheterization OR "balloon catheterization"

#8 thrombectomy OR "mechanical thrombectomy" OR "mechanical thrombolysis" OR "neurothrombectom*" OR "thrombus aspiration" OR "blood vessel prosthesis" OR "blood vessel transplantation" OR "cerebral revascularization" OR reperfusion OR dilatation OR angioplast* OR stent* OR atherect* OR thromboaspiration OR endoluminal repair* OR endovascular snare* OR neuronet OR microsnare OR angiojet OR "penumbra system" OR "solitaire" OR "trevo"

#9 "Retrieval device"[Title/Abstract:~5] OR "Retrieval devices"[Title/Abstract:~5] OR "extraction device"[Title/Abstract:~5] OR "extraction devices"[Title/Abstract:~5] OR "blood vessel prosthesis"[Title/Abstract:~5] OR "blood vessel implantation"[Title/Abstract:~5] OR "artery prosthesis"[Title/Abstract:~5] OR "artery implantation"[Title/Abstract:~5]OR "merci retriever"[Title/Abstract:~5] OR "concentric retriever"[Title/Abstract:~5]

#10 #6 OR #7 OR ##8 OR #9

#11 child OR children OR pediatric* OR paediatric* OR teen* OR Adolescen* OR Youth* OR newborn* OR neonate* OR toddler* OR infant*

#12 "below 18 years" OR "under 18 years" OR "younger than 18"

#13 #11 OR #12

#14 #5 AND #10 AND #13

***WOS:***

**#1** ALL=("cerebrovascular disorders" OR "basal ganglia cerebrovascular disease" OR "carotid artery thrombosis" OR "carotid artery, internal, dissection" OR "cerebral arterial diseases" OR "cerebral sinus thrombosis" OR "cerebral venous sinus thrombosis" OR "CVST" OR "CVT" OR "anterior cerebral artery infarction" OR "middle cerebral artery infarction" OR "posterior cerebral artery infarction" OR "Lacunar Infarction" OR "intracranial embolism" OR "intracranial thrombosis" OR "brain infarction" OR "vertebral artery dissection" OR "brain ischemia")

**#2 TS=((("Ischemi*" OR "Ischaemi*") near/6 ("stroke*" OR "apoplex*" OR "cerebral vasc*" OR "cerebrovasc*" OR cva OR "attack*")))**

**#3 TS=(((brain OR "cerebr*" OR "cerebell*" OR "vertebrobasil*" OR "hemispher*" OR "intracran*" OR intracerebral OR infratentorial OR supratentorial OR "middle cerebr*" OR "anterior circulation") near/5 ("Ischemi*" OR "Ischaemi*" OR "infarct*" OR "thrombo*" OR "emboli*" OR "occlus*" OR "hypoxi*")))**

**#4 #1 OR #2 OR #3**

**#5 ALL=("interventional radiology" OR catheterization OR "balloon catheterization" OR angioplasty OR "laser‐assisted balloon angioplasty" OR "laser angioplasty" OR atherectomy OR "catheter ablation" OR embolectomy OR "aspiration embolectomy" OR "balloon embolectomy")**

#6 ALL=("penumbra system" OR "solitaire" OR "trevo" OR "neurothrombectom*")

#7 ALL=(thrombectomy OR "blood vessel prosthesis" OR "blood vessel transplantation" OR "cerebral revascularization" OR reperfusion OR dilatation OR angioplast* OR stent* OR atherect* OR thromboaspiration OR endoluminal repair* OR endovascular snare* OR neuronet OR microsnare OR angiojet)

#8 TS=((interventional NEAR/3 ("radiolog*" OR "radiograph*" OR "neuroradiolog*")))

#9 TS=(((mechanical OR "radiolog*" OR pharmacomechanical OR laser OR endovascular OR neurovascular) NEAR/5 ("thrombolys*" OR reperfusion OR fragmentation OR aspiration OR recanalisation OR recanalization OR "clot lysis" OR "clot lyses")))

#10 TS=(((clot OR thrombus OR thrombi OR "embol*") NEAR/5 ("aspirat*" OR "remov*" OR "retriev*" OR fragmentation OR "retract*" OR "extract*" OR "obliterat*" OR "dispers*")))

#11 TS=(((retrieval OR extraction) NEAR/5 "device*"))

#12 TS=((("blood vessel" OR artery) NEAR/5 (prosthesis OR "implantat*")))

#13 TS=(((merci OR concentric) NEAR/5 retriever))

#14 TS=(((Endovascular OR intravascular) NEXT ("Procedure*" OR "Technique*" OR "treatment*")))

#15 #5 OR #6 OR #7 OR #8 OR #9 OR #10 OR #11 OR #12 OR #13 OR #14

#16 ALL=(child OR children OR pediatric* OR paediatric* OR teen* OR Adolescen* OR Youth* OR newborn* OR neonate* OR toddler* OR infant*)

#17 ALL=("below 18 years" OR "under 18 years" OR "younger than 18" )

#18 #16 OR #17

#19 #4 AND #15 AND #18

***SCOPUS:***

( TITLE-ABS-KEY ( "cerebrovascular disorders" OR "basal ganglia cerebrovascular disease" OR "brain ischemia" OR "carotid artery diseases" OR "carotid artery thrombosis" OR "carotid artery, internal, dissection" OR "intracranial arterial diseases" OR "cerebral arterial diseases" ) OR TITLE-ABS-KEY ( "anterior cerebral artery infarction" OR "middle cerebral artery infarction" OR "posterior cerebral artery infarction" OR "infarction, anterior cerebral artery" OR "infarction, middle cerebral artery" OR "infarction, posterior cerebral artery" ) OR TITLE-ABS-KEY ( "Lacunar Infarct" OR "Lacunar Infarction" OR "intracranial embolism" OR "intracranial thrombosis" OR "brain infarction" OR "vertebral artery dissection" ) OR TITLE-ABS-KEY ( ( ( brain OR cerebr* OR cerebell* OR vertebrobasil* OR hemispher* OR intracran* OR intracerebral OR infratentorial OR supratentorial OR "middle cerebr*" OR "anterior circulation" ) W/5 ( ischemi* OR ischaemi* OR infarct* OR thrombo* OR emboli* OR occlus* OR hypoxi* ) ) ) OR TITLE-ABS-KEY ( ( ( ischemi* OR ischaemi* ) W/6 ( stroke* OR apoplex* OR "cerebral vasc*" OR cerebrovasc* OR cva OR attack* ) ) ) OR TITLE-ABS-KEY ( "cerebral sinus thrombosis" OR "cerebral venous sinus thrombosis" OR "CVST" OR "CVT" ) )

**AND**

( TITLE-ABS-KEY ( "interventional radiology" OR catheterization OR "balloon catheterization" OR angioplasty OR "laser‐assisted balloon angioplasty" OR "laser angioplasty" OR atherectomy OR "catheter ablation" OR embolectomy OR "aspiration embolectomy" OR "balloon embolectomy" ) OR TITLE-ABS-KEY ( thrombectomy OR "mechanical thrombectomy" OR neurothrombectom* OR "thrombus aspiration" OR "blood vessel prosthesis" OR "blood vessel transplantation" OR "cerebral revascularization" OR reperfusion OR dilatation ) OR TITLE-ABS-KEY ( angioplast* OR stent* OR atherect* OR thromboaspiration OR endoluminal AND repair* OR endovascular AND snare* OR neuronet OR microsnare OR angiojet OR "penumbra system" OR "solitaire" OR "trevo" ) OR TITLE-ABS-KEY ( ( interventional W/3 ( radiolog* OR radiograph* OR neuroradiolog* ) ) ) OR TITLE-ABS-KEY ( ( ( mechanical OR radiolog* OR pharmacomechanical OR laser OR endovascular OR neurovascular ) W/5 ( thrombolys* OR reperfusion OR fragmentation OR aspiration OR recanalisation OR recanalization OR "clot lysis" OR "clot lyses" ) ) ) OR TITLE-ABS-KEY ( ( ( clot OR thrombus OR thrombi OR embol* ) W/5 ( aspirat* OR remov* OR retriev* OR fragmentation OR retract* OR extract* OR obliterat* OR dispers* ) ) ) OR TITLE-ABS-KEY ( ( ( retrieval OR extraction ) W/5 device* ) ) OR TITLE-ABS-KEY ( ( ( "blood vessel" OR artery ) W/5 ( prosthesis OR implantat* ) ) ) OR TITLE-ABS-KEY ( ( ( merci OR concentric ) near/5 AND retriever ) ) OR TITLE-ABS-KEY ( ( ( endovascular OR intravascular ) W/2 ( procedure* OR technique* OR treatment* ) ) ) )

**AND**

( TITLE-ABS-KEY ( child OR children OR pediatric* OR paediatric* OR teen* OR adolescen* OR youth* OR newborn* OR neonate* OR toddler* OR infant* ) OR TITLE-ABS-KEY ( "below 18 years" OR "under 18 years" OR "younger than 18" ) )

**
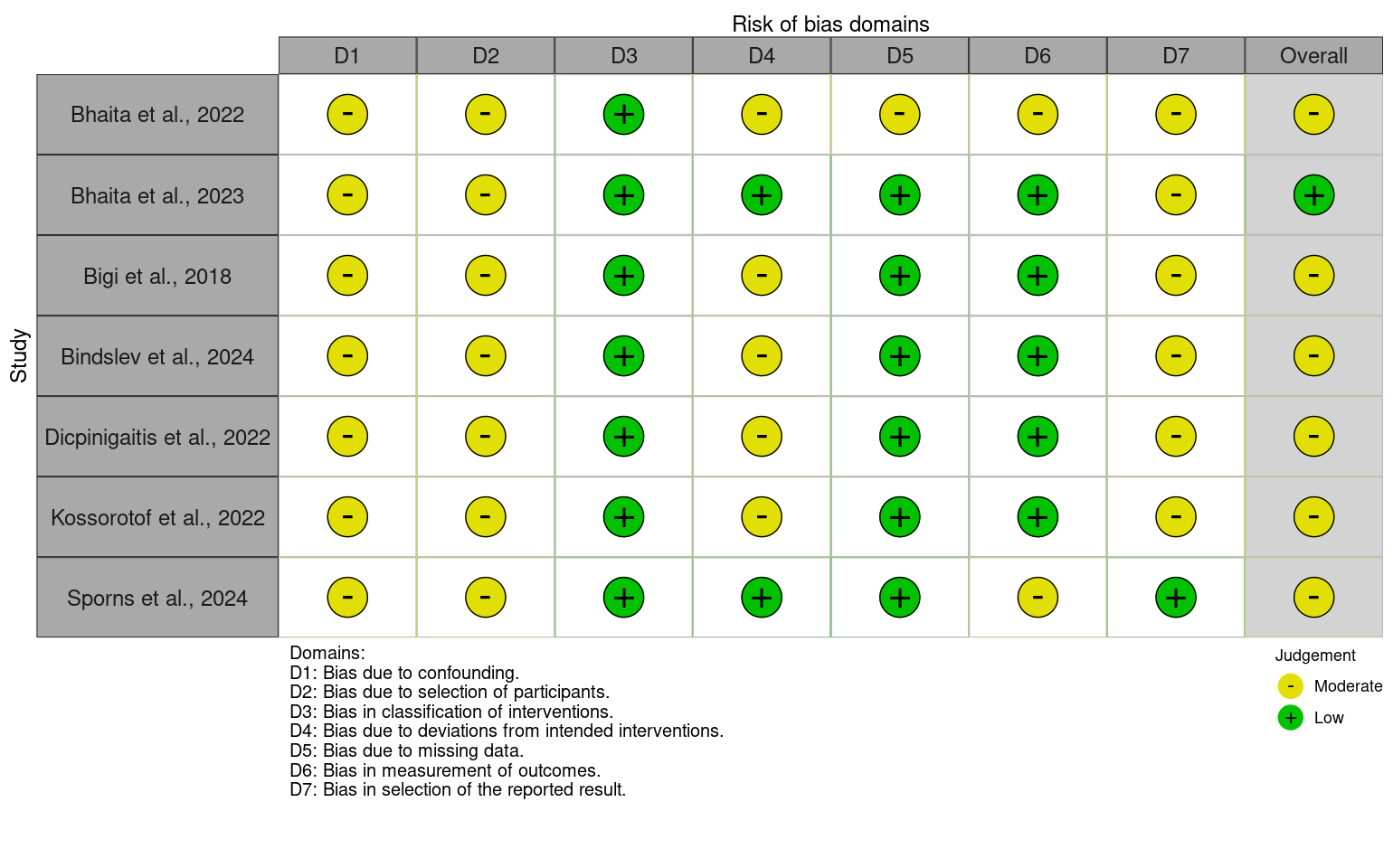
Figure. S1 Traffic Light Plot of Risk of Bias Assessment Using the ROBINS-E Tool**

**
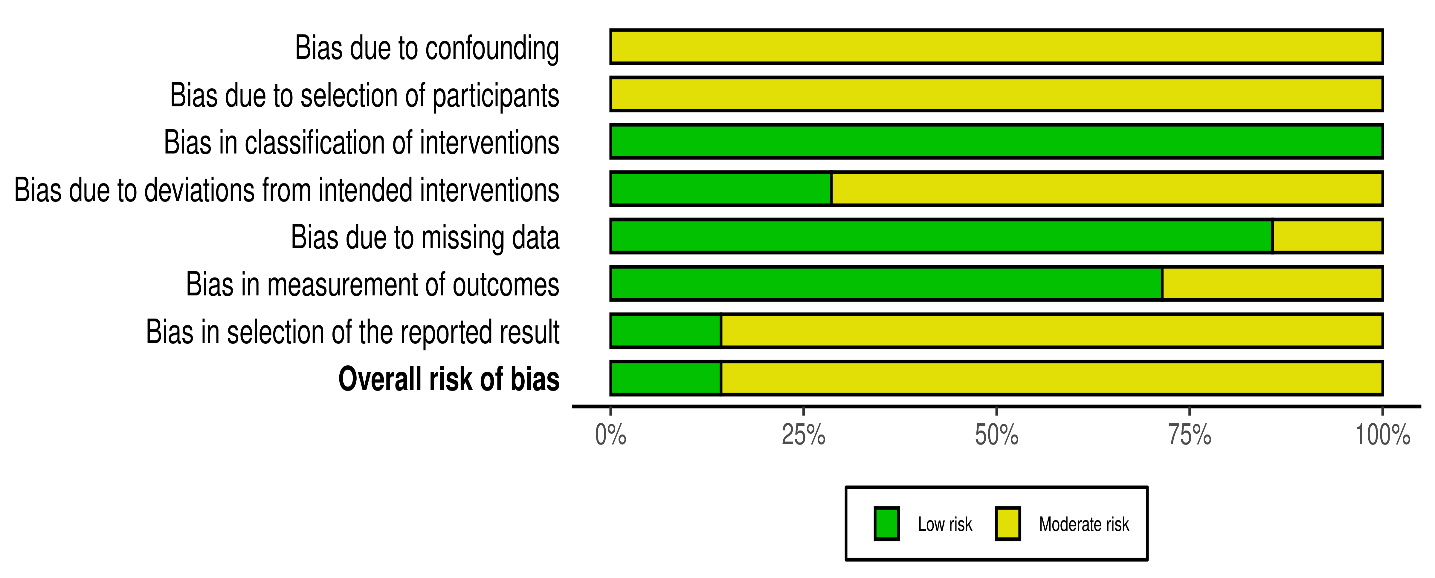
**

**Figure. S2 Summary Plot of Risk of Bias Assessment Using the ROBINS-E Tool**


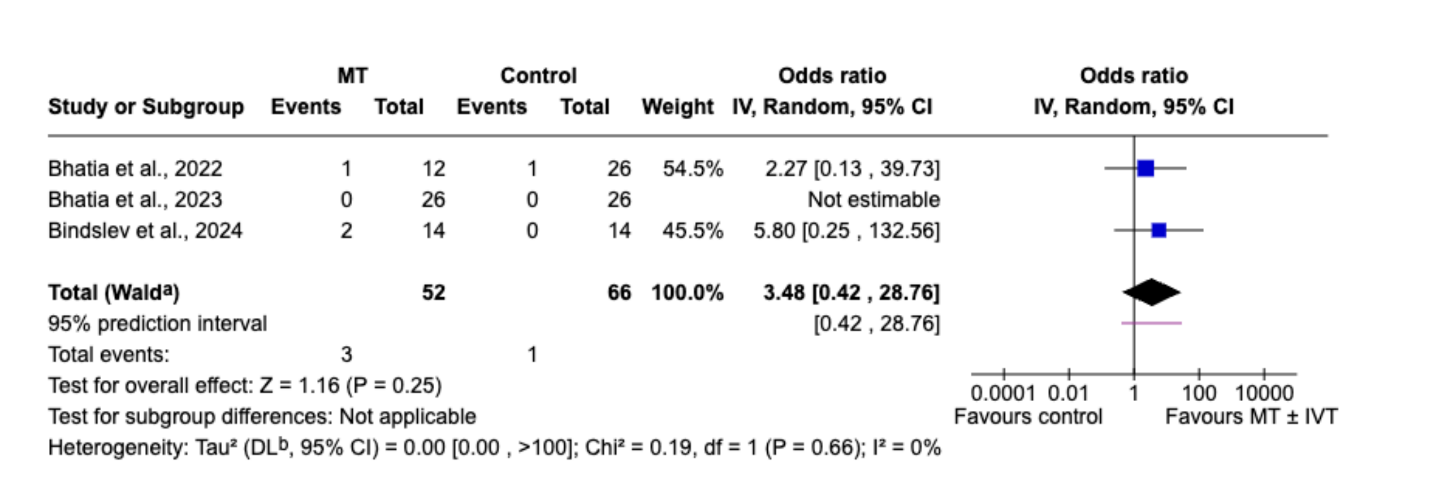


**Figure S3.** Forest plot for mortality in patient with large vessel occlusion stroke.


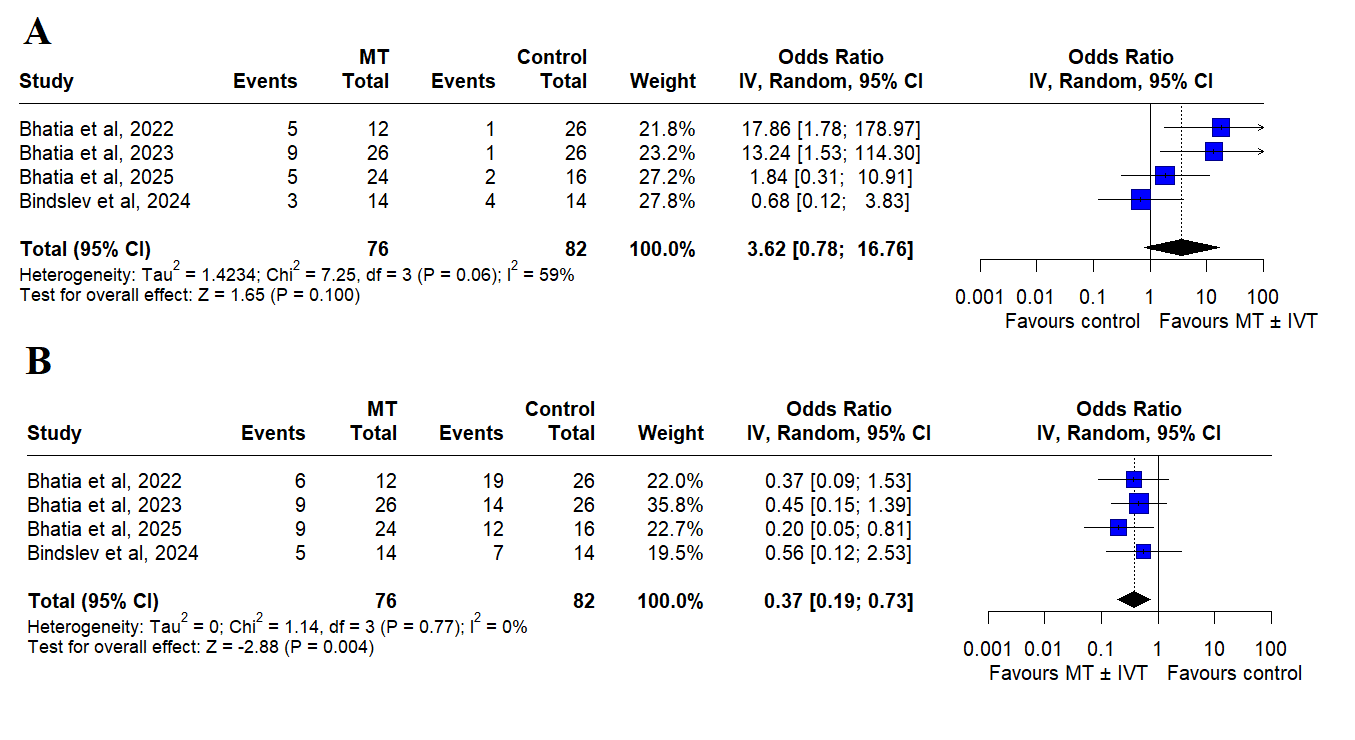


**Figure S4.** Forest plots for (a) excellent functional recovery, and (b) poor functional recovery in patient with large vessel occlusion stroke
